# The Elementary Operations of Human Vision Are Not Reducible to Template-Matching

**DOI:** 10.1371/journal.pcbi.1004499

**Published:** 2015-11-10

**Authors:** Peter Neri

**Affiliations:** Laboratoire des Systèmes Perceptifs, CNRS UMR 8248, 29 rue d’Ulm, 75005 Paris, France; Institut d’Etude de la Cognition, Ecole Normale Supèrieure - PSL Research University, 75005 Paris, France; University of York, UNITED KINGDOM

## Abstract

It is generally acknowledged that biological vision presents nonlinear characteristics, yet linear filtering accounts of visual processing are ubiquitous. The template-matching operation implemented by the linear-nonlinear cascade (linear filter followed by static nonlinearity) is the most widely adopted computational tool in systems neuroscience. This simple model achieves remarkable explanatory power while retaining analytical tractability, potentially extending its reach to a wide range of systems and levels in sensory processing. The extent of its applicability to human behaviour, however, remains unclear. Because sensory stimuli possess multiple attributes (e.g. position, orientation, size), the issue of applicability may be asked by considering each attribute one at a time in relation to a family of linear-nonlinear models, or by considering all attributes collectively in relation to a specified implementation of the linear-nonlinear cascade. We demonstrate that human visual processing can operate under conditions that are indistinguishable from linear-nonlinear transduction with respect to substantially different stimulus attributes of a uniquely specified target signal with associated behavioural task. However, no specific implementation of a linear-nonlinear cascade is able to account for the entire collection of results across attributes; a satisfactory account at this level requires the introduction of a small gain-control circuit, resulting in a model that no longer belongs to the linear-nonlinear family. Our results inform and constrain efforts at obtaining and interpreting comprehensive characterizations of the human sensory process by demonstrating its inescapably nonlinear nature, even under conditions that have been painstakingly fine-tuned to facilitate template-matching behaviour and to produce results that, at some level of inspection, do conform to linear filtering predictions. They also suggest that compliance with linear transduction may be the targeted outcome of carefully crafted nonlinear circuits, rather than default behaviour exhibited by basic components.

## Author Summary

Any attempt to model human vision must first ask: can it be approximated by a process that linearly matches the visual stimulus with an internal template? We often take this approximation for granted without properly checking its validity. Even if we assume that the approximation is valid under specific conditions, does this mean the system operates template matching across the board? We would not know exactly in what sense and to what extent the approximation may be viable. Our results address both issues. We find that template matchers are locally applicable in relation to a wide range of conditions, providing much-needed justification for several relevant computational tools. We also find, however, that there is no sense in which the system is globally a linear template: it remains inescapably nonlinear. Our findings suggest that linear transduction is not cost-free: it is not a default building block that is used for constructing expensive nonlinear processes. Rather, linear sensory representations arise from carefully constructed nonlinear processes that strike a balanced act between the necessity to retain other important computations, and the desirability of transducing and representing the visual world on a linear scale.

## Introduction

Animals constantly submit environmental signals to neural operations designed to extract useful information for guiding behaviour. Whether their sensory apparatus is considered in its entirety as a behavioural machine or in relation to hardware components like individual nerve cells, it can be described as an input-output transformation that maps external stimuli onto neural representations. The simplest way to characterize the operation of such a sensory device is to assign a set of weights to different elements of the incoming stimulation, then sum across all elements, and finally convert this weighted sum into a number compatible with the scale and units of the output variable [1, 2]. For input stimulus **s**, this simple operation can be written as *g*(⟨**s**,**w**⟩) (**w** is the weighting function, ⟨, ⟩ inner product, and *g* a static nonlinearity). To provide an example, **w** may be the receptive field of a simple cell, and *g* the nonlinearity that maps membrane voltage onto average spike rate [3]. For another example, more relevant to the present study, we can think of **w** as the perceptual impact associated with different portions of a visual display presented to a human observer, and *g* the decisional transducer that maps aggregate perceptual impact onto a binary decision of the kind ‘I saw the stimulus’ or ‘I did not see it’ [4].

The above linear-nonlinear cascade model has been applied to innumerable phenomena in neuroscience [5, 6], to the extent that it would be impossible to summarize them here. Particularly when referring to perceptual processes, it is often termed ‘template-matching’ [7] to indicate that an internal template (the filter) is matched against the incoming stimulus (via linear weighting) before a decision is made as to whether the stimulus does or does not contain the template signal [8, 9]. We will use the terms ‘linear-nonlinear’ (abbreviated LN) and ‘template matcher’ interchangeably. We will also occasionally refer to this process as ‘linear filtering’ or ‘linear transduction’ and contrapose it to a ‘nonlinear’ process, with the understanding that in these instances we are specifically referring to the processing stage that precedes the decisional nonlinearity (*g* in the previous paragraph). The latter element is an integral part of all psychophysical models (and those considered here are no exception), but can be largely bypassed to access the preceding layers using the methods employed in this study [4, 10].

Qualitative thinking about sensory processing almost invariably refers back to the LN model [11], not least because its explanatory scope can be greatly extended by adopting arbitrary descriptors for **s**, effectively remapping the stimulus onto a space that is available for inner-product treatment [12]. The well-known dipper function for contrast discrimination, for example, is often accounted for by a specific choice of *g* for a human observer modelled as a LN cascade [13], and the link to neural activity can also be inferred via this simple framework [14]. Apparently counter-intuitive phenomena such as stochastic resonance can be accommodated via the LN model [15]. Furthermore, certain methodological approaches (notably reverse correlation) often rely on the assumption that the system of interest is well approximated by the LN operator [4, 16, 17].

Notwithstanding such widespread applicability, there are well-known instances when the LN model is unable to provide a satisfactory account of relevant phenomena. The operation of a complex cell, for example, cannot be described by the LN cascade acting directly on the stimulus image [18]. Several neural systems exhibit pronounced gain control properties [19], and these too fall outside the explanatory reach of barebone LN operators. In human vision, detection under uncertainty represents a classic example of the inapplicability of simple template-matching models belonging to the LN family [20, 21]. Adaptive phenomena, e.g. learning-mediated plasticity, can only be partially approximated by LN descriptors [10]. It is therefore uncontroversial that LN models are sometimes inadequate.

The critical issue is to recognize when they are adequate or not and, whenever they appear adequate for a specific application of the system under interest, how far their applicability can be generalized to other applications of that same system. We can illustrate this issue with the following example. In experiment 1, we characterize the response of a neuron, or a whole observer, to visual orientation by selectively manipulating the orientation content of a simple visual stimulus (e.g. a textured object; see [22] for an example from literature). In experiment 2, we characterize the response of the same system to pattern size by selectively manipulating the spatial frequency (SF) content of the same stimulus (see [23] for examples). First, we ask whether the manner in which the system operates under the conditions of experiment 1 can be approximated by the LN operator applied to the input stimulus defined with respect to orientation content: **s** is a vector specifying orientation energy in the stimulus for different orientations, and **w** is the orientation tuning function of the system; is *g*(⟨**s**,**w**⟩) adequate? We can ask the same question with reference to experiment 2, except **s** is now the vector specifying stimulus energy across SF, and **w** is the SF tuning function of the system.

An altogether different question is to ask whether the operation of the system with relation to *both* orientation and SF can be accounted for by the same LN cascade [24, 25]. For this purpose, the visual stimulus must be projected onto a space **s** that encompasses both orientation and SF, because the LN cascade must be applied to one common input space; at the same time, it must be able to make predictions for the two different spaces probed in the two separate experiments. The natural space of choice in this case is that of the image itself, i.e. the 2D pixel array detailing stimulus intensity at each spatial location on the display. If we call this image ${}^{\text{2D}}\text{s}$, the question is whether we can identify linear filter ${}^{\text{2D}}\text{w}$ and nonlinear transducer *g* so that $g(\langle^{\text{2D}}\text{s}{,}^{\text{2D}}\text{w}\rangle )$ will capture the results of experiments 1 and 2. As we demonstrate in this study, a positive answer to the question posed in the previous paragraph (i.e. both experiments falling within the explanatory power of the LN family) does not guarantee a positive answer to this latter question: the system may appear to operate in the manner of the LN cascade with relation to a number of different probes defined within substantially different spaces (orientation, SF, 2D space), yet its behaviour may not be collectively captured by a single LN cascade. Our results have important implications for the applicability of LN cascades to visual perception, and establish some general notions/tools relating to both the potential and limitation of this modelling family for capturing human sensory processing.

## Methods

### Ethics statement

Ethics approval was obtained from the College Ethics Review Board (CERB) at Aberdeen University (http://www.abdn.ac.uk/clsm/working-here/cerb.php). All participants gave written informed consent.

### Stimuli and task specifications

**Fig 1:**
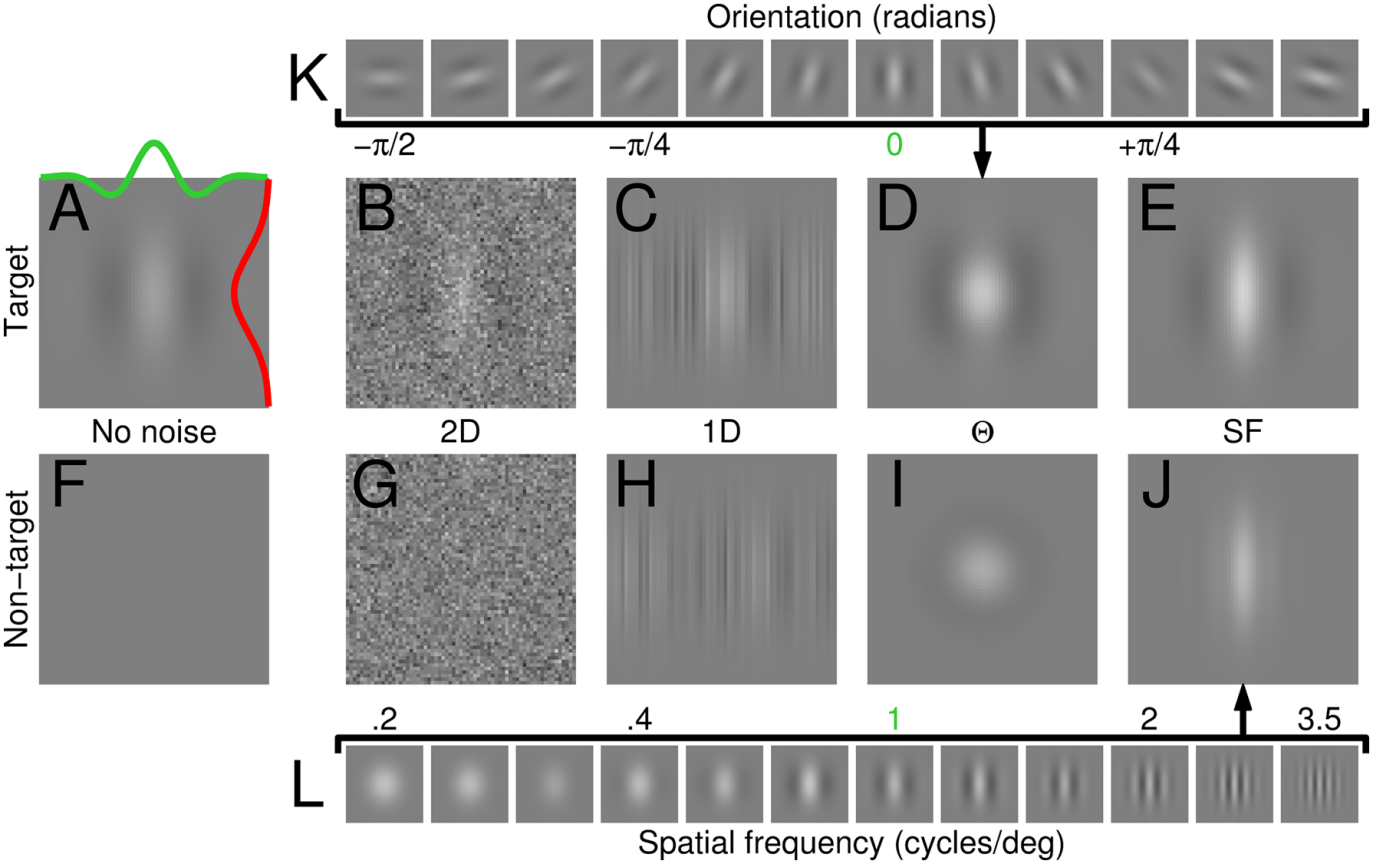
Same signal but different noise. Observers were asked to discriminate a vertical Gabor target signal (A) from a non-target signal (blank in F). Four different noise types were added to both target and non-target signals. In the 2D noise condition (B,G) each pixel was assigned a random Gaussian modulation. In the 1D condition (C,H) noise only varied along the horizontal dimension in the form of bar-like Gaussian modulations. In the orientation (Θ) condition (D,I), noise consisted of the sum of a set of Gabor patches spanning the entire orientation range (K), each patch taking on a randomly assigned contrast value (see Methods). Spatial frequency (SF) noise (E,J) was generated using a similar procedure, except the underlying patch set varied across SF (L) rather than orientation. The green (alternatively red) profile in A displays a horizontal (alternatively vertical) slice through the target surface. Green labels in K (alternatively L) point to target location along Θ (alternatively SF) dimensions.

**Fig 2:**
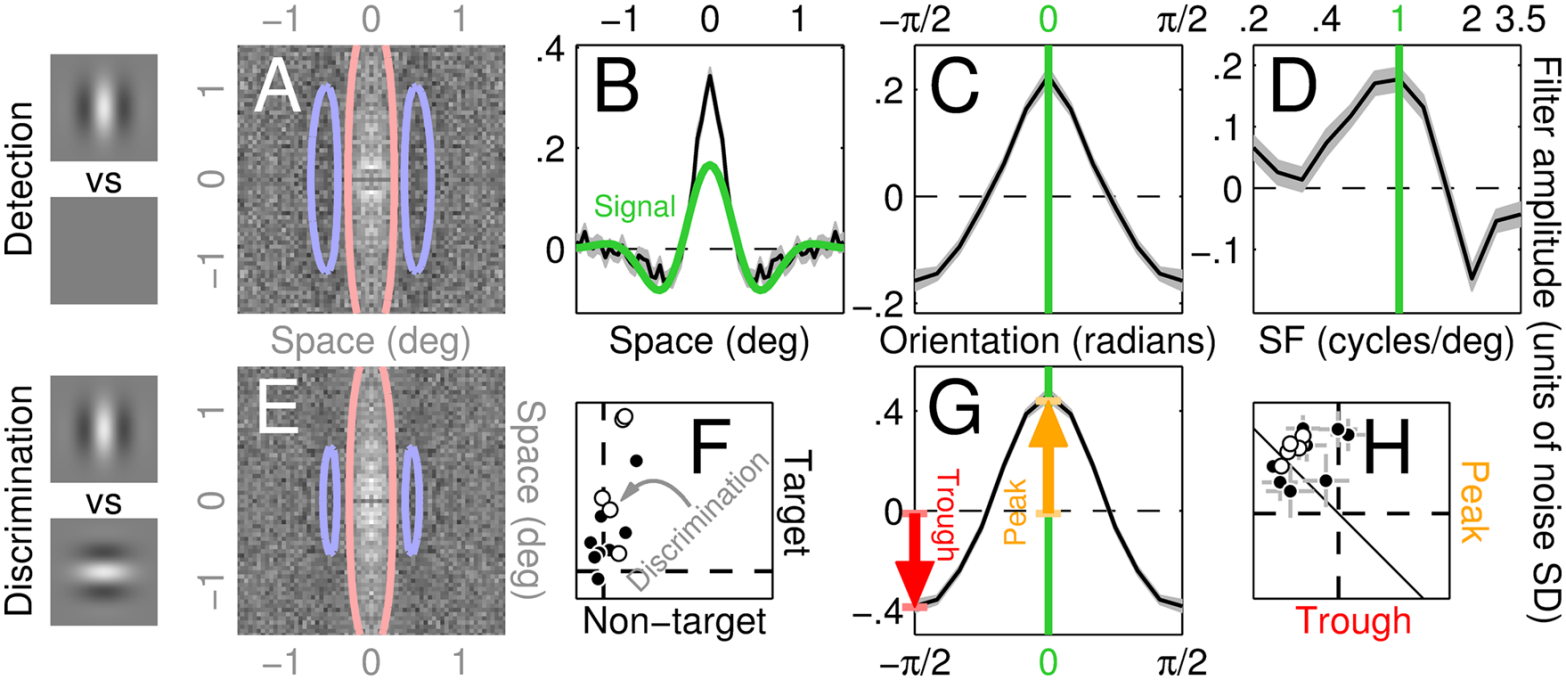
Perceptual filters used by human observers to detect/discriminate stimuli. A-D show aggregate (across observers) perceptual filters (PF) for detecting the vertical Gabor target (icons to the left of A) returned by reverse correlating different noise types: 2D (A), 1D (B), Θ (C) and SF (D; see Fig 1 for image samples of all four classes). Red/blue lines in A show positive/negative contours through a Gabor fit to the data. Green trace in B and green lines in C-D indicate target signal. Grey shaded regions in B-D show ±1 SEM. Panels E,G show results corresponding to A,C (2D and Θ noise probes) for discriminating vertical from horizontal Gabor targets (icons to the left of E). F plots match between 2D PF’s and target/non-target signals on y/x axes (non-target as specified in the discrimination task) for both detection (solid) and discrimination (open) across different observers (different data points). H plots filter amplitude of orientation-tuned PF’s (C,G) at 0 (peak, y axis) and ±π/2 (trough) using similar conventions. Error bars (±1 SEM) in F,H are sometimes smaller than symbols and therefore not visible. The centre value of 2D PF’s (A,E) is effectively undefined because occluded by the fixation marker (see Methods).

Stimuli lasted 80 ms and consisted of 3 regions (∼3×3 deg each): a central ‘probe’ region at fixation (the fixation marker consisted of a dark pixel in the centre measuring ∼3×3 arcmin and never disappeared); above and below it, two identical ‘reference’ regions containing the template (see S1 Video). The template signal consisted of a cosine-phase (peaking at centre) vertical Gabor wavelet (standard deviation (SD) of Gaussian envelope 0.5 deg, spatial frequency 1 cycles/deg), and was presented at 17% contrast within the reference regions (background luminance 30 cd/m^2^). On each trial, observers saw two instances of the stimulus separated by a 500-ms gap. Reference regions were identical on both instances (and across all trials), thus providing no useful information for performing the task; their purpose was to remind observers of the target signal shape, so as to facilitate a template-matching strategy [21, 26]. The probe region contained target signal plus noise mask on one instance, and non-target signal plus noise mask on the other instance. Observers were asked to select the instance (first or second) that contained the target signal by pressing one of two buttons, after which they received immediate trial-by-trial feedback (correct/incorrect). The target signal was simply the template Gabor wavelet described above (see also Fig 1A), presented at 8% (alternatively 4%) contrast in the detection (alternatively discrimination) task. The non-target signal was blank for the detection task (Fig 1F), and a horizontal variant of the target signal for the discrimination task (see icons to the left of Fig 2E). Except for taking on a different orientation, the non-target signal in the discrimination task was identical to the target-signal. Data for the two tasks were collected in separate blocks of 100 trials each. We also collected separate data for a ‘symmetric’ variant of the discrimination task. In this additional experiment, two identical reference regions containing non-target templates were presented to the left and to the right of the central probe region.

### Noise masks

**Fig 3:**
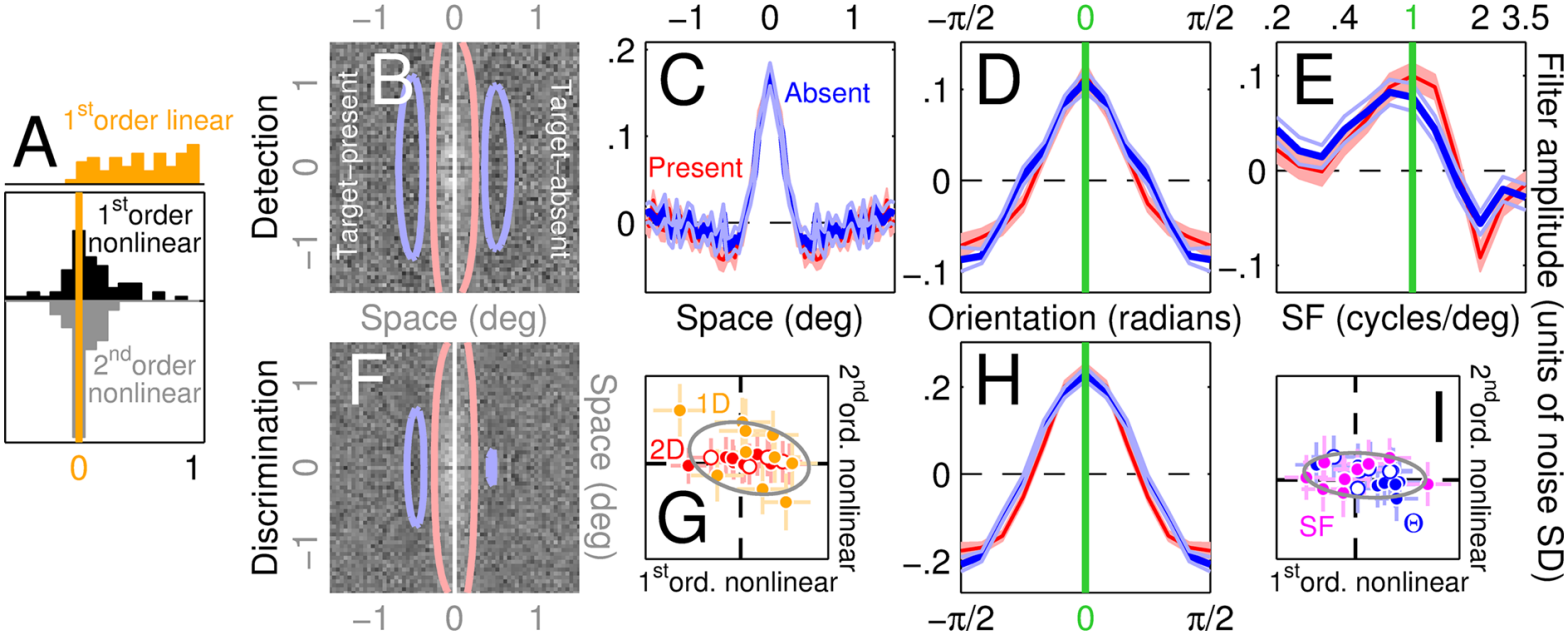
Nonlinear tests return no deviations, i.e. compliance with template matching. Panels B-F,H plot the same as Fig 2A–2E and 2G but separately for target-present and target-absent PF’s (see Methods). The two descriptors match, as predicted by the linear-nonlinear (LN) cascade. The 1^st^-order nonlinear test is designed to quantify potential differences between the two first-order descriptors (see Methods). G plots the outcome of this test for individual observers (one data point per observer per condition) on the x axis for the 2D (red, solid/open for detection/discrimination) and 1D (orange) conditions, versus the outcome of the 2^nd^-order nonlinear test designed to quantify potential modulations within second-order PF’s (these should be featureless for the LN cascade [10]). Both tests scatter around 0 (indicated by dashed lines). I plots the same for Θ (blue) and SF (magenta) conditions. Error bars in G,I show ±1 SEM; gray ovals are centred on mean across all data points, with axes tilted along linear fit and spanning ±2 SD of projected data values. A plots distributions for both tests across all conditions/observers, together with a similar descriptor (orange) quantifying modulations within full first-order PF’s (i.e. computed from all trials; this additional analysis is presented here to demonstrate that the adopted test metric is sensitive to structure when present).

**Fig 4:**
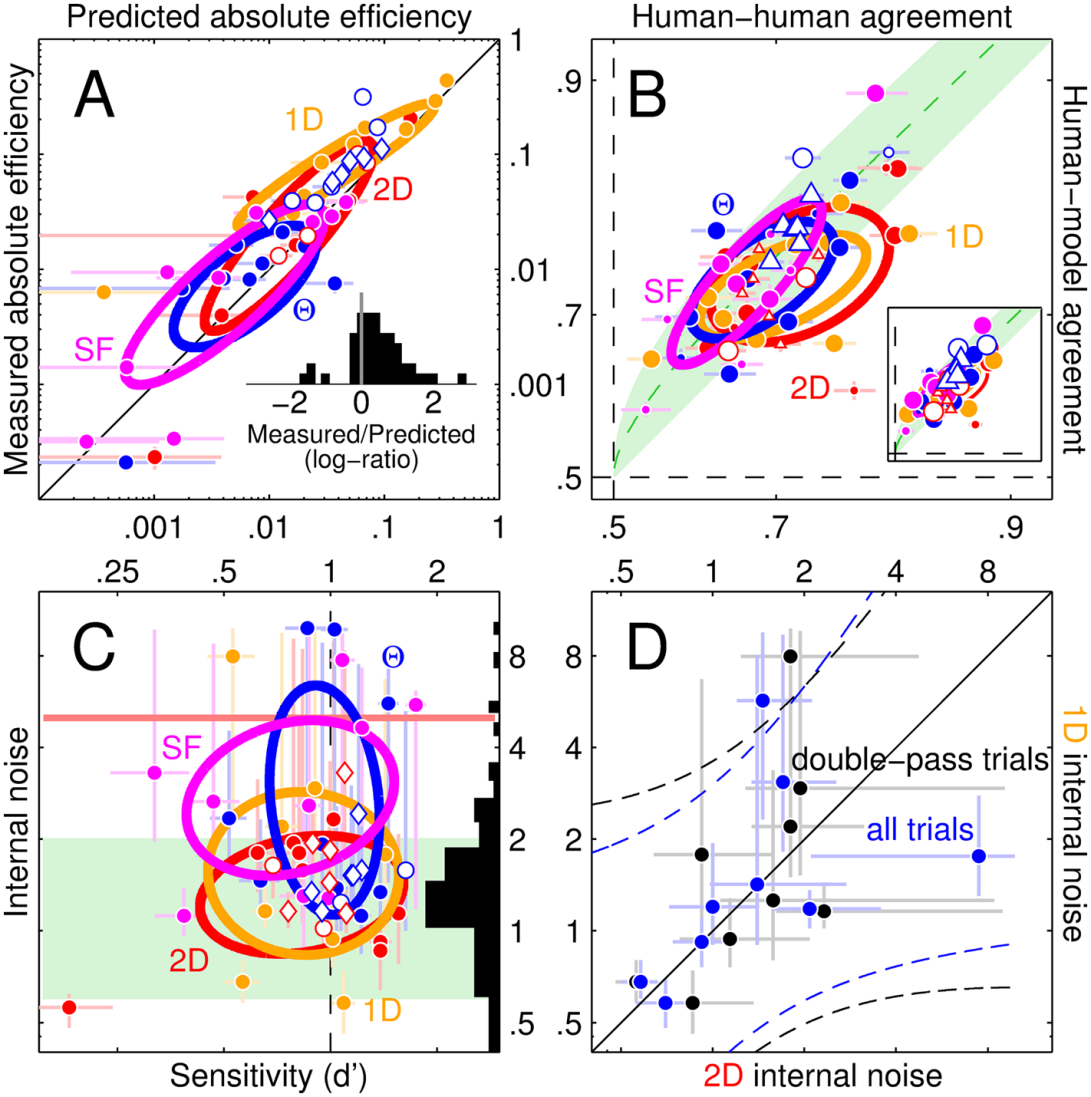
Human intrinsic variability is compatible with a linear model followed by a late additive noise source. The absolute efficiency predicted by the LN model (x axis in A) matches corresponding human estimates (y axis). Inset to A plots distribution of log-ratios between measured and predicted values (0 when not different). Trial-by-trial agreement between human responses and LN model (y axis in B) falls within the optimal range (indicated by green shading; green dotted line shows midpoint between upper and lower boundaries); inset shows similar results for gain-control model (diagram in Fig 6A). Symbol size in B reflects associated decoupled baseline (small size for baseline≤chance, big for baseline>chance; see S1 Text for details on how baseline was computed). Axes have been stretched to map linearly in d′ units. Internal noise estimates (see Methods) plotted on y axis in C (see also side histogram) are broadly consistent with measurements from previous studies [27] (range indicated by green shading) and do not correlate with sensitivity (x axis), as expected of an additive noise source. Red horizontal line marks cut-off point (value of 5) beyond which internal noise estimates represent estimation failures [27]. Estimates for 2D (x axis in D) versus 1D noise from the detection task are mildly correlated across observers (dashed lines show ±95% confidence intervals on linear fit) but do not differ (data points scatter around solid unity line), consistent with a late common noise source (an earlier noise source may be expected to scale with noise dimensionality [28]). Black/blue symbols in D show estimates obtained by computing the percentage of correct responses from double-pass blocks only (black) or all blocks (blue) to demonstrate that this choice had little impact on the resulting trend. In all remaining panels, solid/open symbols refer to detection/discrimination tasks and different colours refer to different noise types (red for 2D, yellow for 1D, blue for orientation, magenta for SF). Diamond shape refers to the symmetric variant of the discrimination experiment. Each symbol refers to data from an individual observer in the condition specified by its colour/shape characteristics. Error bars show ±1 SEM in all plots (omitted in inset to B to avoid clutter); ovals are centred on mean across data points, with axes tilted along linear fit and spanning ±1 SD of projected data values.

The noise mask could be 1 of 4 different types in the detection task, and 1 of 2 different types in the discrimination task (thus explaining why the detection data in Figs 2B and 2D and 3C and 3E are not matched by equivalent data for discrimination). Mask type was randomly selected on every trial with equal probability for each mask. In the detection task, the noise mask could be ‘2D’ (Fig 1B and 1G): each pixel (within a 65×65 array) was separately assigned a random luminance value from a zero-mean Gaussian distribution with SD ∼16% contrast (we use the ∼ symbol because this value was tailored to each observer to target threshold performance of d′ ∼1, see abscissa values in Fig 4C); ‘1D’ (Fig 1C and 1H): each column of pixels spanning the probe region was separately assigned a random value from a zero-mean Gaussian distribution with SD ∼9% contrast, and the vertical profile of each column was modulated by the envelope (Gaussian window) of the Gabor target signal; ‘**Θ**’ (Fig 1D and 1I): a set of 12 Gabor wavelets spanning the 0-*π* orientation range (Fig 1K), all identical to the target signal except for rotation, were assigned a random contrast value from a Gaussian distribution with mean ∼3% (alternatively ∼5%) contrast and SD ∼0.7% (alternatively ∼1.2%) contrast in the detection (alternatively discrimination) task; ‘SF’ (Fig 1E and J): a set of 12 Gabor wavelets ranging in spatial frequency (SF) from 0.2 to 3.5 cycles/deg in logarithmic steps (Fig 1L), all identical to the target signal except for SF, were assigned a random contrast value from a Gaussian distribution with mean ∼3% contrast and SD ∼0.7% contrast. Noise masks were specified as detailed above so that each mask type was associated with a non-zero probability of realizing the target signal. Because only 2D and Θ masks present a non-zero probability of realizing the non-target signal in the discrimination task, only these two masks were adopted for the discrimination condition. Furthermore, due to their vertical characteristic and the limited contrast range afforded by a combination of design and hardware constraints, 1D and SF noise probes did not effectively mask the horizontal non-target signal and introduced spurious cues (e.g. cross-oriented regions) for performing the task. 2D/1D noise was fully orthogonal (each pixel was modulated independently and did not overlap with other pixels); Θ/SF noise was not fully orthogonal because the underlying wavelets (Fig 1K–1L) were not themselves orthogonal except for specific instances. In relation to the logic of this study (see Results) orthogonality was important only between vertical and horizontal components of orientation noise (indicated by peak and trough respectively in Fig 2G–2H); these two components were very nearly orthogonal (within hardware precision). All aspects of the study were validated via explicit implementation of fully specified computational models (see below).

### Observers and data mass

We tested 10 observers in the detection task (∼3.3K trials per noise type per observer, total of ∼130K trials); 5 of those 10 observers also participated in the discrimination task (∼4.4K trials per noise type per observer, total of ∼44K trials), and 1 of those 10 observers also participated in the symmetric variant of the discrimination task together with an additional 5 observers who did not belong to the original pool of 10 observers (∼4K trials per noise type per observer, total of ∼49K trials). All observers were naive to the purpose and methodology of the study; they were paid 7GBP/9EUR per hour for their participation. The total number of trials collected for this study (single-pass and double-pass, see below for description of the latter type) was 269700.

### Perceptual filter (PF) computation

Following completion of data collection and acquisition of observer responses, we can classify each stimulus ${}^{\text{type}}z^{\left[q,r\right]}$ as being of type type presented in the target-present (*q* = 1) or target-absent (*q* = 0) interval on a specific trial, to which observers responded correctly (*r* = 1) or incorrectly (*r* = 0). It is a matrix of dimension 65×65 for type 2D, a 65-element vector for type 1D, a 12-element vector for type Θ and SF. It was constructed by summing a signal s to a noisy sample n: ${}^{\text{type}}z^{\left[q,r\right]}={}^{\text{type}}s^{\left[q\right]}+{}^{\text{type}}n^{\left[q,r\right]}$. s and n were specified as detailed above. When projected onto 2D space s is the same across type (see Fig 1), however its vector representation with respect to each type differs: it is the Gabor wavelet specified above for type 2D, a horizontal slice through said wavelet for type 1D (green trace in Fig 2B), a non-zero entry for the 7th element of a 12-element vector for type Θ and SF. The first-order target-present PF (i.e. computed only from noise fields containing the target) was ${}^{\text{type}}{\text{p}}_{1}^{\left[1\right]}=\text{avg}({}^{\text{type}}n^{\left[1,1\right]})-\text{avg}({}^{\text{type}}n^{\left[1,0\right]})$ where avg(.) indicates average across the subset of trials indexed by the assigned type and [*q*, *r*] values [4]; the target-absent PF was ${}^{\text{type}}{\text{p}}_{1}^{\left[0\right]}=\text{avg}({}^{\text{type}}n^{\left[0,0\right]})-\text{avg}({}^{\text{type}}n^{\left[0,1\right]})$. The full PF was simply the sum of target-present and target-absent PF’s: ${}^{\text{type}}{\text{p}}_{1}={}^{\text{type}}p_{1}^{\left[1\right]}+{}^{\text{type}}p_{1}^{\left[0\right]}$ [29]. The second-order PF is similarly computed as ${}^{\text{type}}p_{2}=\text{cov}{(}^{\text{type}}n^{\left[1,1\right]})+\text{cov}{(}^{\text{type}}n^{\left[0,0\right]})-\text{cov}{(}^{\text{type}}n^{\left[1,0\right]})-\text{cov}{(}^{\text{type}}n^{\left[0,1\right]})$ where cov(.) indicates covariance across trials of the specified classification [10].

### Scalar metrics applied to PF

Because we found an inevitable degree of variability across observers, it is difficult to draw conclusions from simply inspecting individual PF’s. We therefore performed additional analyses that captured relevant aspects of filter structure, and quantified each aspect using a single value (scalar metric) for each PF. This approach made it then possible to perform simple population statistics and confirm or reject specific hypotheses (against unambiguously defined null benchmarks) about the overall shape of the filters. Our conclusions are therefore based on individual observer data, not on the aggregate observer; aggregate descriptors (e.g. Fig 2A–2E and 2G) are only presented for visualization purposes. Some previous studies using classified noise have relied on qualitative inspection of aggregate data, but this approach is inadequate to draw robust conclusions primarily for two reasons: 1) there is no generally accepted procedure for generating an average PF from individual images for different observers [30]; 2) we have shown in previous work that effects observed via qualitative inspection of aggregate filters may not survive quantitative inspection using metric analysis, and vice versa [22].

#### Match with target signal

For ${}^{\text{2D}}p_{1}$, $\langle^{\text{2D}}p_{1}{,}^{\text{2D}}s^{\left[q\right]}\rangle$ is the match with target (*q* = 1, plotted on y axis in Fig 2F) or non-target signal (*q* = 0, plotted on x axis in Fig 2F) used in the discrimination task (icons to the left of Fig 2E); ⟨, ⟩ is Frobenius (2D) inner product (element-by-element multiplication followed by sum across all elements).

#### Test of structure presence within first-order/second-order PF’s

We computed two PF descriptors separately from odd and even trials and calculated their element-by-element correlation. The expected value of this cross-validated correlation is 0 for structureless PF’s, because the two descriptors are computed from independent noise samples; any departure from 0 reflects the presence of measurable structure that was available from non-overlapping subsets of the data. We then exploited this measure to test for two predictions of LN transduction: 1) target-present PF’s must match target-absent PF’s [21, 31–38]; 2) second-order PF’s must be featureless [10]. To test for the first prediction (referred to as ‘1^st^-order nonlinear’ test because it is based on 1^st^-order PF’s), the cross-validated procedure was applied to the difference between target-present and target-absent PF’s. To test for the second prediction (referred to as ‘2^nd^-order nonlinear’ test), it was applied to second-order PF’s. We adopted a variant of the cross-validation procedure in the 2D case, due to the large dimensionality of the associated PF and the resulting reduction in resolving power of our estimates: by computing element-by-element correlation across the entire 2D PF, we may have missed existing structure due to insufficient resolution of our measurements. We therefore reduced the dimensionality of the PF descriptor by restricting it to the two marginal averages across the PF surface, resulting in a PF descriptor of size 65+65. We also verified that our conclusions were unaffected by the adoption of marginalization procedures other than average, such as root-mean-square (RMS). For the 2^nd^-order nonlinear test applied to the 2D case, we only used the diagonal (variance as opposed to covariance) of the second-order PF (the dimensionality of the full 2D second-order PF (65^4^) being impracticably large for reliable estimation). We often plot results from the two tests in the form of scattergraphs (e.g. Fig 3G and 3I) to expose potential correlations, because the quantities measured by the two tests are linked by theoretical considerations [10] (Fig 8I offers examples of both correlated and uncorrelated characteristics).

### Response agreement and internal noise estimation

We performed a series of additional experiments specifically designed to measure human-human agreement [39]. During these experimental blocks, observers saw the same stimuli presented under typical data collection conditions, with the only difference that the second half of each 100-trial block (from trial #51 to trial #100) consisted of a repetition of the first half (from trial #1 to trial #50) in randomly permuted order. Human-human agreement (plotted on the x axis in Fig 4A) is simply the % of repeated trials on which observers gave the same response. By adopting a minimal signal detection theory (SDT) framework, internal noise (plotted on the y axis in Fig 4C) can be estimated (in units of external noise) from human-human agreement and the % of correct-response trials. Details of this routine procedure have been extensively documented in previous publications [27, 39, 40]. Human-model agreement (plotted on the y axis in Fig 4B) is the % of trials on which the human response matches the response generated by a computational model to the same stimulus set (see below for details of models implemented here). For a given value of human-human agreement *x* in a 2AFC task, upper and lower bounds on the maximum achievable human-model agreement are given by $(1+\sqrt{2x-1})/2$ and *x* itself [41]; the corresponding region is indicated by green shading in Fig 4B. Human-model agreement may exceed chance for models that are decoupled from the trial-by-trial perturbation delivered by the external noise source; to identify instances where this may be the case, we have developed a ‘decoupled’ baseline (see S1 Text). We collected double-pass data from 9 of the 10 observers in the detection task (∼650 trials per noise type per observer, total of ∼23K trials), 2 of the 5 observers in the discrimination task (∼1050 trials per noise type per observer, total of ∼4.2K trials), and all 6 observers in the symmetric variant of the discrimination task (∼1650 trials per noise type per observer, total of ∼20K trials). The first half/pass of this dataset was combined with the original dataset for the purpose of PF estimation and associated analysis.

### Computational models

#### Template matcher (LN)

**Fig 5:**
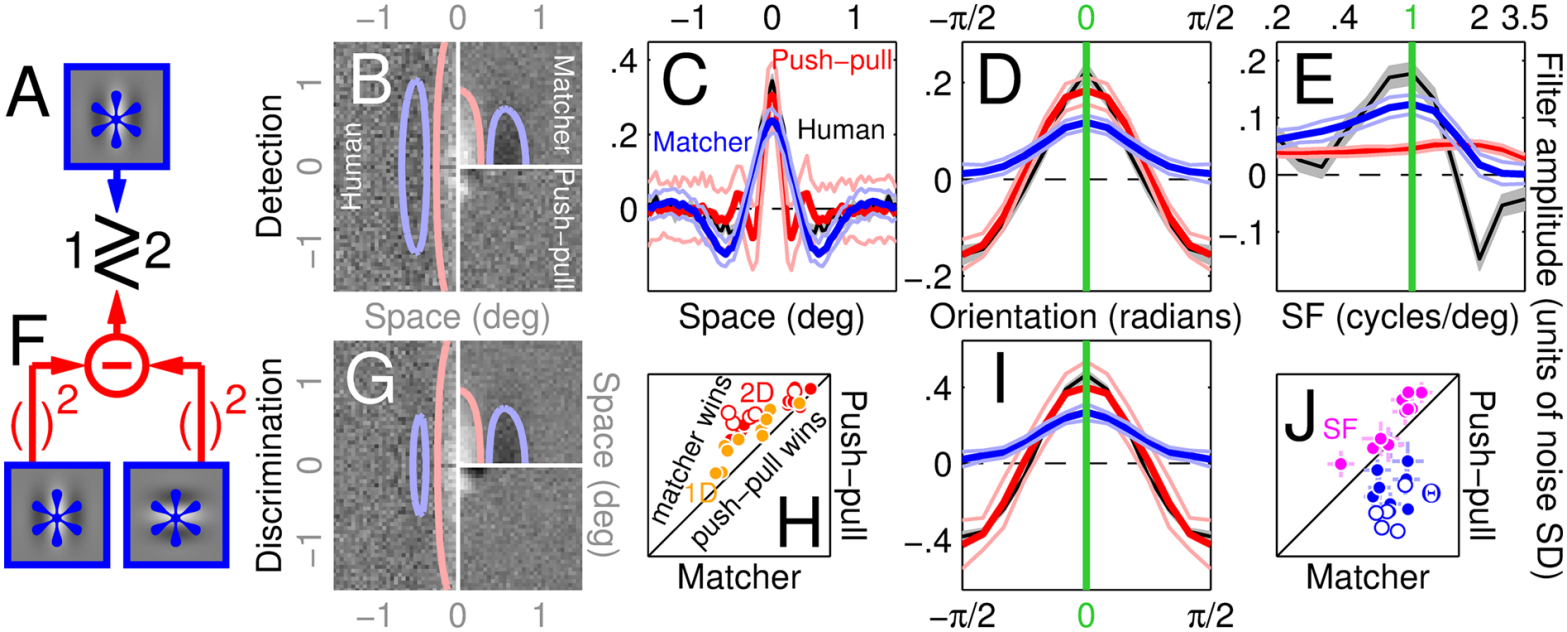
No single LN model is able to account for the entire dataset. B-E,G,I plot human PF’s shown in Fig 2A–2E and 2G together with simulated PF’s returned by a single 2D implementation of the matcher (LN) model (blue in C-E,I) or the push-pull model (red in C-E,I; see diagrams in A,F for schematics of the two models). Error regions around simulated PF’s show ±1 SD across simulations. H,J show normalized root-mean-square distance (see Methods) between simulated and human PF’s for matcher model (x axis) versus push-pull model (y axis) using the plotting conventions adopted in Fig 3G and 3I.

We implemented three different models (plus opponent variants for two of them), which we refer to as matcher, push-pull and gain-control. In the matcher model, each stimulus $z^{\left[q\right]}$ is matched (via inner product) to template t, obtaining response $r^{\left[q\right]}=\langle z^{\left[q\right]},t\rangle$. The model responds correctly if the difference between target and non-target match, *r* ^[1]^ − *r* ^[0]^, is positive; it responds incorrectly otherwise. This decisional rule was adopted for all models (in line with current literature [42]). The matcher model was applied across all 4 stimulus spaces explored in the experiments, with ${}^{\text{type}}t$ equal to the estimated PF ${}^{\text{type}}p_{1}$ for the given type; corresponding human-model agreement is plotted on the y axis in Fig 4B (main panel). For the purpose of computing human-model agreement from this model, we exploited symmetry to increase PF reliability (e.g. we mirror-averaged 2D PF’s across vertical and horizontal axes). We also implemented a 2D variant (diagram in Fig 5A) where ${}^{\text{2D}}t$ matched the target signal ${}^{\text{2D}}s^{\left[1\right]}$ and all stimuli were projected to 2D before being subjected to the template; the corresponding PF’s across noise types are shown in Fig 5 (blue traces in panels C-E,I).

#### Push-pull

We only implemented the 2D variant of the push-pull model (diagram in Fig 5F). Each input stimulus is matched to both the target signal and to its orhtogonal image ${}^{\text{2D}}s^{\left[0\right]}$ (the non-target signal in the discrimination task, see icons to the left of Fig 2E); the model response associated with the stimulus is the difference between the two squared matches: $r^{\left[q\right]}=\langle^{\text{2D}}z^{\left[q\right]}{,}^{\text{2D}}s^{\left[1\right]}\rangle {}^{2}-\langle^{\text{2D}}z^{\left[q\right]}{,}^{\text{2D}}s^{\left[0\right]}\rangle {}^{2}$. The corresponding PF’s across noise types are shown in Fig 5 (red traces in panels C-E,I). The corresponding results for 1^st^- and 2^nd^-order nonlinear tests are plotted in Fig 7B.

#### Gain-control

**Fig 6:**
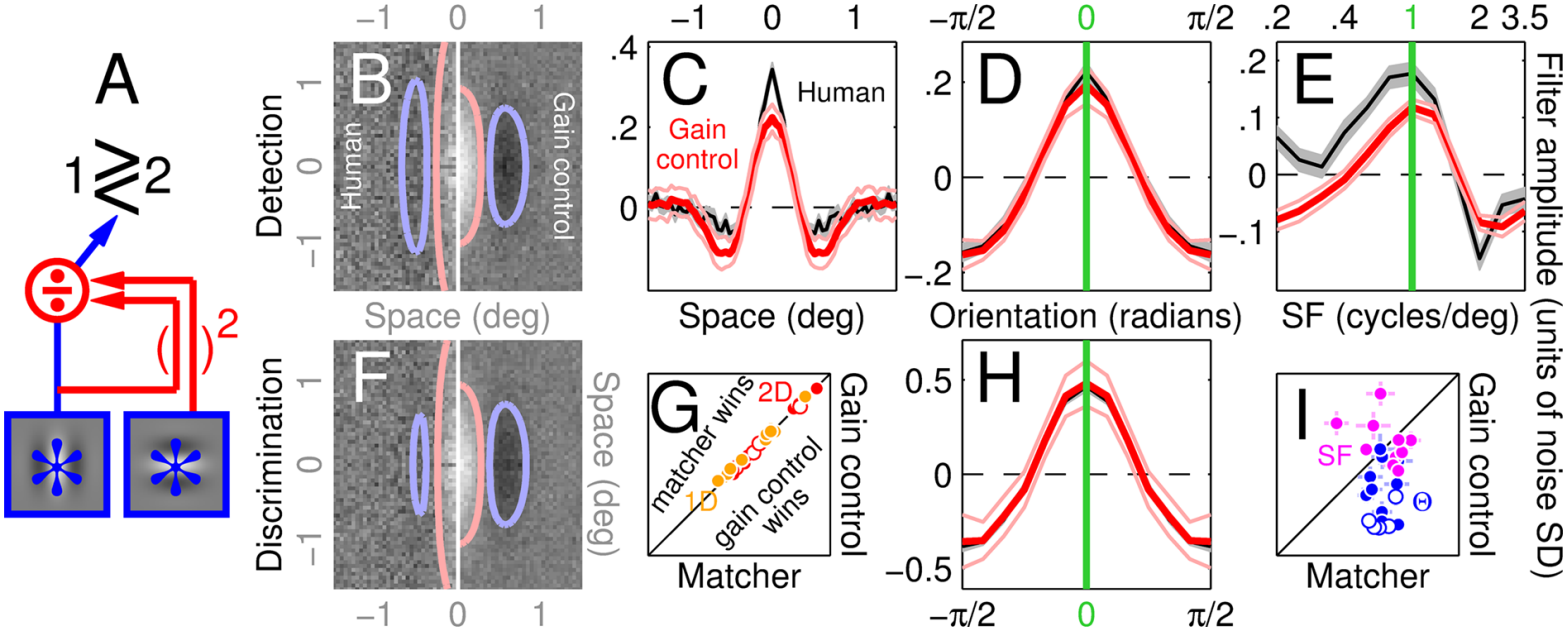
Gain-control model accounts for the entire dataset. Plotting conventions as in Fig 5. Diagram in A summarizes structure of the gain-control model that generated the PF’s in B-F,H.

**Fig 7:**
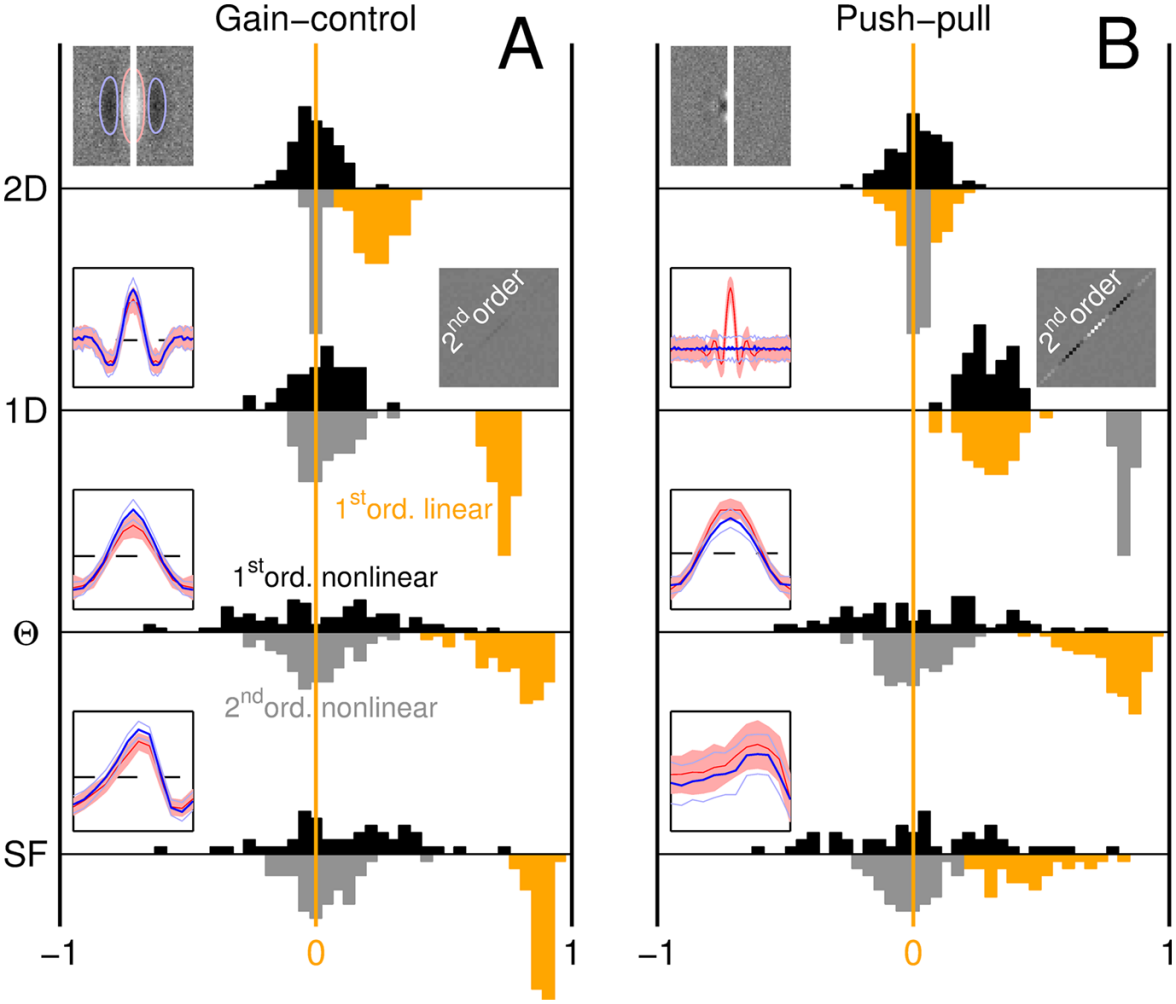
Nonlinear tests return no deviations for gain-control model, but detect nonlinear behaviour exhibited by push-pull model. Black histograms plot distributions for the 1^st^-order nonlinear test, grey histograms for the 2^nd^-order nonlinear test (see caption to Fig 3 for brief description of these two tests), orange histograms for 1^st^-order full PF’s (reflecting linear component of LN models when applicable), across all 4 noise conditions (different rows) and for both gain-control (A) and push-pull model (B). Left insets plot target-present/target-absent (red/blue) first-order PF’s (similar to Fig 3B–3E); right insets plot second-order PF’s for the 1D condition. The second-order PF associated with the push-pull model displays substantial modulations; in the 1D condition, this model returns clearly positive values for both nonlinear tests. All simulated first-order PF’s display measurable structure (see orange distributions) except for the push-pull model in the 2D condition (top right, see also Fig 5B and 5G).

We only implemented the 2D variant of the gain-control model (diagram in Fig 5F). The model response associated with stimulus ${}^{\text{2D}}z^{\left[q\right]}$ is given by the output of the matcher model detailed earlier with template equal to the target signal, divided by a normalizing factor *k*: $r^{\left[q\right]}=\langle^{\text{2D}}z^{\left[q\right]}{,}^{\text{2D}}s^{\left[1\right]}\rangle /k$. *k* was obtained by the Pythagorean sum of the two matches detailed above for the push-pull model (rather than their difference): $k={\left(\langle^{\text{2D}}z^{\left[q\right]}{,}^{\text{2D}}s^{\left[1\right]}\rangle {}^{2}+\langle^{\text{2D}}z^{\left[q\right]}{,}^{\text{2D}}s^{\left[0\right]}\rangle {}^{2}\right)}^{\frac{1}{2}}$. Human-model agreement for this model is plotted on the y axis in the inset to Fig 4B; the associated PF’s across noise types are shown in Fig 6 (red traces in panels C-E,H). The corresponding results for 1^st^- and 2^nd^-order nonlinear tests are plotted in Fig 7A.

#### Opponent variants

**Fig 8:**
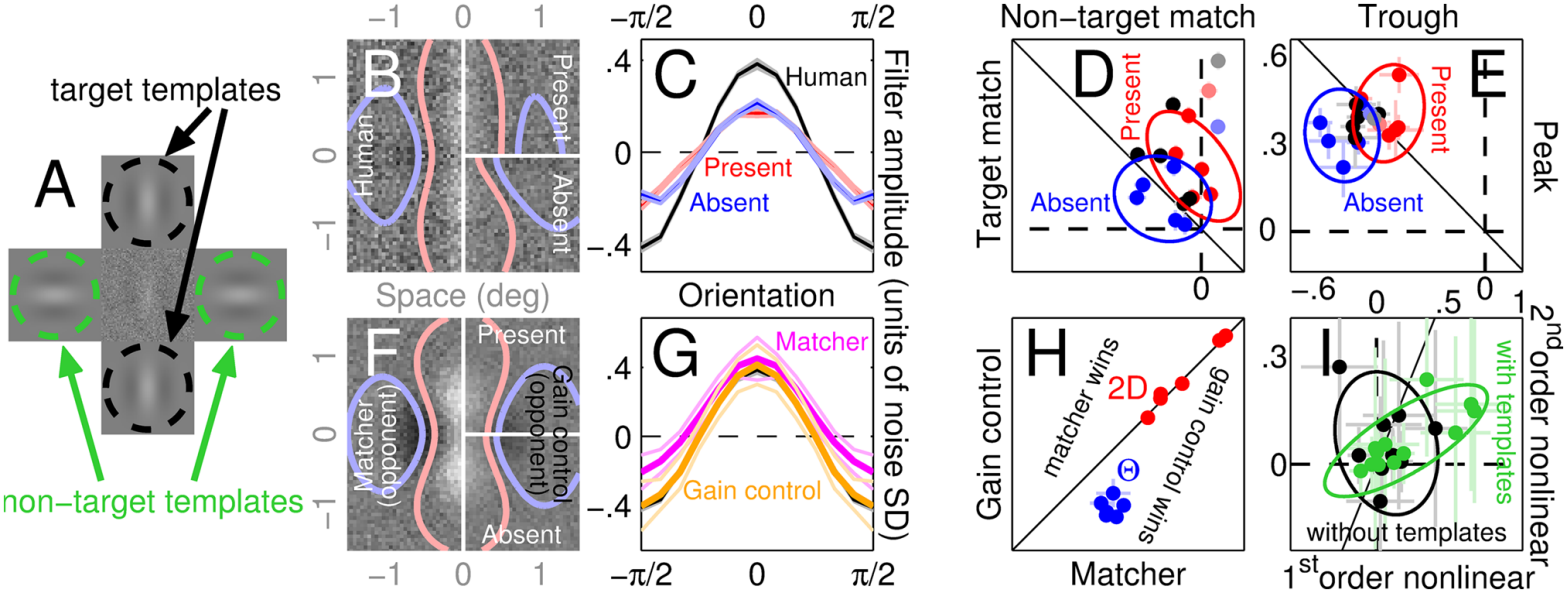
Symmetric variant of discrimination task engages two elementary operations with orthogonal preference. The central probe was flanked by additional non-target templates (indicated by green in A). B-C plot overall (labelled ‘Human’) as well as target-present/absent PF’s for 2D and Θ conditions (similar plotting conventions to Fig 3B and 3D). F plots the overall 2D PF from the opponent variant of the LN model (left) and target-present/absent PF’s from the opponent variant of the gain congrol model (right; see Methods for detailed description of these variants). G plots overall Θ PF’s from these two models (magenta and yellow respectively) together with the human PF replotted from C (black). D plots target/non-target match values (similar to Fig 2F) from overall (black), target-present (red) and target-absent (blue) 2D PF’s; light colouring refers to the observer who had also participated in the original discrimination experiment without non-target templates. E plots peak/trough amplitudes of Θ PF’s (similar to Fig 2H) using similar colour-coding conventions. H plots normalized root-mean-square distance between simulated and human PF’s using the conventions adopted in Figs 5H and 5J and 6G and 6H. I plots outcome of 1^st^- and 2^nd^-order nonlinear tests using the conventions of Fig 3G and 3I across observers and noise masks from the original discrimination experiment (black) and its symmetric variant (green).

In the opponent variants of the template-matcher and gain-control models, two units like those described above were combined via subtraction. We call *o* ^[1]^ the output of the vertically-oriented unit detailed above (whether template-macther or gain-control), and *o* ^[0]^ the output of a horizontally-oriented unit; the two units are identical except for the different orientation of the linear drive. The output of the opponent variant is $r^{\left[q\right]}=o^{\left[1\right]}-\frac{1}{2}o^{\left[0\right]}$ (we halved *o* ^[0]^ because 2D PF’s from the symmetric variant of the discrimination task displayed ∼2×more vertical than horizontal structure, see black symbols in Fig 8D).

#### Late additive internal noise source

For the stimulus SNR’s used with human observers, model outputs were often always correct (or nearly so). Under those conditions it becomes impossible to apply PF estimation. We therefore introduced a late additive noise source to the simulations, intended to approximate the internal noise source intrinsic to human observers [27] (see above). The differential output *r* ^[1]^ − *r* ^[0]^ from the model was added to Laplace-distributed noise with shape and scale parameters set to unity [26] before generating a binary choice.

#### Normalized root-mean-square distance from simulated PF

We computed the RMS value of the difference between human and simulated PF’s after separately rescaling each to RMS = 1. Rescaling was necessary because PF amplitude depends on several factors [10], notably internal noise [17]. Although in this study we were able to measure internal noise magnitude for individual observers (see previous section), the resulting estimates often presented a substantial degree of experimental error and variability (see vertical error bars in Fig 4C), making it impractical to incorporate them into our models. Furthermore, the impact of internal noise on PF amplitude is well understood only for a late additive source [10, 17], while our goal in estimating distance between human and simulated PF’s was to retain validity regardless of the internal noise process that was affecting human performance. For the 2D case, prior to computing RMS distance we reduced PF dimensionality using the procedure already described in the previous section.

## Results

### Empirical estimates of human perceptual filters

We asked observers to detect the most common target stimulus in contemporary vision science [43]: the Gabor wavelet (Fig 1A). On each trial, one interval contained this target signal, while the other interval did not (Fig 1F); observers were asked to select the target interval. We then added four different types of visual noise to both target and non-target stimuli: 2D pixel noise (Fig 1B and 1G), 1D ‘line’ noise (Fig 1C and 1H), orientation (Fig 1D and 1I) and spatial frequency (SF) noise (Fig 1E and 1J). We applied psychophysical reverse correlation [10, 17] to retrieve the perceptual filters (PF) associated with each noise probe separately (see Methods). The PF can be thought of as the psychophysical equivalent of the physiological receptive field [11, 41]: it provides an overall picture of the weighting function applied by the observer to the incoming stimulus for the purpose of identifying the assigned target signal [4, 17]. This description is useful for intuitive purposes, but is inaccurate and possibly misleading upon closer inspection due to important differences between the two processes instantiated by single neurons on the one hand, and human observers on the other [10, 41].

The different noise probes were randomly mixed within the same block, so that observers did not know which noise type would appear on the next trial (see S1 Video); furthermore, their task was identical throughout all blocks, regardless of the noise type applied on any given trial. Data from different noise types therefore enable different vantage points on the same underlying elementary visual operation (extraction of a localized oriented wavelet). As expected, the PF’s associated with different noise types resemble the target signal: the 2D spatial PF presents a Gabor-like modulation not dissimilar from the target (compare Fig 2A with Fig 1A), the 1D spatial PF takes on a Mexican-hat shape that largely overlaps with a horizontal slice through the target (compare black data with green trace in Fig 2B), the orientation-tuned PF peaks at target orientation (indicated by green line in Fig 2C), and the SF-tuned PF peaks at target SF (indicated by green line in Fig 2D).

In a series of additional experiments, we determined that similar results were obtained for a discrimination variant of the same task where the non-target signal was orthogonal to the target signal (icons to the left of Fig 2E). The PF’s associated with the discrimination task were virtually identical to those returned by the detection task (compare E with A and G with C in Fig 2; only 2D and Θ masks are applicable to this task, see Methods), contrary to the ideal observer prediction that the 2D PF should be an image of the target minus the non-target signal [1]: the estimated PF for discrimination (Fig 2E) appears to contain exclusively vertically oriented structure (across observers and detection/discrimination tasks, PF match with target (vertically-oriented) on y axis in Fig 2F is significant at p < 10^−3^ (Bonferroni-corrected for multiple comparison) by two-tailed Wilcoxon signed rank (WSR) test for match>0, while match with rotated target (horizontally oriented) on x axis is not significant at p>0.05). A feature of specific interest for the purposes of later modelling efforts is that orientation-tuned PF’s (Fig 2C and 2G) present clear troughs at the non-target orientation (±*π*/2 on x axis) of magnitude comparable to their peaks (across observers/tasks, peak/trough amplitudes on y/x axes in Fig 2H modulate significantly at p < 10^−3^ (same test as above) and are not different (except for sign) at p>0.05 by paired two-tailed WSR test). This result is not trivially expected as other outcomes are possible (see [22] for examples).

The lack of any discernible difference between discrimination and detection experiments indicates that the same mechanism supports both operations, prompting us to seek a single model able to account for the entire dataset in Fig 2.

### Applicability of LN cascades

We carried out 4 tests, based on established literature [10, 34, 36, 38, 39, 41, 44], designed to gauge the applicability of LN transduction. They converge to indicate that, for any given representation of the visual stimulus, the LN framework provides an adequate description of the manner in which the human sensory system operates with respect to that representation.

The first test capitalizes on the prediction by the LN model that filter estimates returned by noise fields associated with the target stimulus (target-present) must match estimates returned by target-absent noise fields [21, 31–38]. We observed virtually no difference between the two estimates (compare left versus right surface plots in Fig 3B and 3F and blue versus red traces in C-E,H), consistent with the prediction of the LN model (see below for individual observer analysis and quantitative corroboration of the above-detailed qualitative observations).

An additional test relies on nonlinear (second-order) operators that capture system properties not conveyed by first-order estimates [33] (see Methods); the LN model predicts structureless second-order perceptual filters [10]. Fig 3G and 3I plot an index of such second-order nonlinear structure (y axis) versus a similar index of structural differences between target-present and target-absent PF’s (relating to the ‘first-order nonlinear’ test described in the previous paragraph) across observers (different data points); neither was significantly different from 0 (p>0.05 whether corrected or not for multiple comparison, one-tailed WSR test; see also overall black/gray distributions in Fig 3A, alongside orange distribution of same structural index applied to overall first-order PF’s to demonstrate that this index can adequately expose filter structure when present). Although the two tests are not equivalent in that second-order nonlinearities do not necessarily impact target-present filter estimates [10], they probe related aspects of the underlying process [26]; they may therefore be expected to correlate in the event of departures from template matching (we provide one such example from data in Fig 8I). Contrary to this expectation, there was no detectable correlation (p>0.05, robust correlation toolbox [45]) between the two tests (see lack of substantial tilt for gray ovals in Fig 3G and 3I), further supporting the notion that if any departure from template matching was present in our data, it was too small to measure.

A third test compares human absolute efficiency (plotted on y axis in Fig 4A) against corresponding predictions for LN models incorporating the empirically estimated PF [44]. There was reasonable agreement between measurements and predictions (points scatter around diagonal unity line), however we report an appreciable tendency for measured efficiency values to exceed corresponding predictions (this effect is visually demonstrated by the tendency for data points to fall above the unity line in Fig 4A; see also histogram within inset). More specifically, when different noise conditions are tested separately, only the 1D condition approaches statistical significance (two-tailed paired WSR test for measured versus predicted estimates returns p < 0.005 but this value does not survive Bonferroni correction for multiple comparison; the remaining three noise conditions return p>0.05 whether corrected or not). When tested collectively across conditions and tasks (all data points in Fig 4A collapsed onto one dataset), measured values exceed predicted values at p < 10^−3^ (two-tailed paired WSR test, survives correction for multiple comparison). Similar deviations have been previously documented for a variety of stimuli and discrimination tasks [44]. Our current understanding of relevant phenomena does not allow us to confidently identify the source of this small discrepancy, and it should be noted that the efficiency predictions are based on specific assumptions about the nature of the internal noise source [44]. The additional test detailed below makes no such assumptions.

The last test compares the output generated by the LN model on specific trials against the human output on those same specific trials [46] (this comparison was also independently assessed with respect to decoupled baseline, see Methods and symbol size in Fig 4B). The resulting human-model agreement (proportion of trials on which the two outputs match) is plotted in Fig 4B (y axis) against the proportion of trials on which human observers replicate their own response for two passes of the same stimulus (human-human agreement, see Methods). Collectively, estimates fall significantly above the lower bound of the maximum predictability region [41] (green shaded area) on a targeted comparison (two-tailed paired WSR test for y>x returns p <10^−5^), they do not differ from the midpoint between upper and lower bound (indicated by the green dotted line in Fig 4B) at p = 0.26, but they are smaller than the upper bound (p < 10^−8^). These findings indicate that the level of trial-by-trial predictability achieved by the LN model is compatible with optimality, although they do not guarantee this result: it remains possible that the internal noise process operating within observers is such that maximum predictability should be assigned to the upper bound of the green area in Fig 4B [41], in which case the trial-by-trial predictability achieved by the LN model would be suboptimal. We currently lack effective tools for characterizing the detailed structure of internal noise within human observers, however based on recent attempts [26] it appears reasonable that the typical region of maximum predictability for human vision should lie between the two extremes; this region is compatible with the values generated by the LN model (see above). We also converted these measurements into estimates of internal noise [39] to confirm that its properties are compatible with the notion of a late additive source often assumed by LN models [4] (Fig 4C–4D, see caption).

It may appear surprising that human visual processing displayed such compliance with the LN model, particularly with relation to the target-present/target-absent comparison (Fig 3): a representative survey of relevant literature indicates that these two estimates differ at least as often as they do not [4, 21, 36], and sometimes substantially so [34, 37, 38, 47]. It may therefore be argued on the basis of pevious studies that the lack of any difference, rather than its presence, should be viewed as unexpected and atypical. Our results, however, must be interpreted in light of the consideration that every aspect of the adopted experimental design was optimized to achieve template matching on the part of human observers (e.g. the stimulus was presented centrally [26], we placed two noiseless target signals above and below the central probe [48], we explicitly instructed observers to match the probe against those target replicas [21, 26], observers were given trial-by-trial feedback). Our objective was to test the applicability of LN modelling under conditions that favoured this processing mode, so that we could gauge the full extent of its explanatory power. Our analyses support the applicability of the LN cascade with respect to each dimension we probed. This result does not imply that the same LN model operating within a common representation also accounts for all results obtained across different representations. We turn to the latter issue below.

### One model for all feature spaces: a small gain-control circuit accounts for all results

In attempting to identify a single specific implementation of the LN cascade that may account for our entire dataset, the natural starting point is a LN model that applies a 2D template similar to the estimated 2D PF (followed by the static decisional nonlinearity, see Methods). We implemented the 2D LN model using the target signal as template (Fig 5A) rather than the estimated PF (Fig 2A). This choice is motivated by the following considerations: 1) it allows us to exploit the full resolving power of the entire dataset by avoiding the need for cross-validation [49]; 2) our results will serve future investigations even where data mass is insufficient to support PF estimation [50]; 3) model evaluation will be based on ‘structural failures’ of the associated simulations (i.e. qualitative departures from data that cannot be ameliorated by tweaking model parameters), so that fine details of template structure are irrelevant.

Unsurprisingly, the 2D LN model generates good PF predictions for 2D/1D probes (Fig 5B–5C), however it is unable to simulate the negative modulations orthogonal to target orientation observed for orientation-tuned PF’s (compare blue with black traces in Fig 5D and 5I); as we have demonstrated in Fig 2H via quantitative analysis, those modulations reflect genuine structure in the data. No degree of model tweaking would allow the 2D LN model to generate those negative modulations: the underlying human template contains no structure along the orientation orthogonal to the target (Fig 2F), which in turn implies orientation-tuned PF’s lacking modulations within that region of orientation space. The LN model specified above must therefore be rejected as a viable account of our dataset. Because this conclusion is solely based on characteristics associated with the linear filtering stage (L) in the LN model (those characteristics are evaluated via the corresponding PF estimates which, under the LN model, return an image of the linear filtering stage), it is valid regardless of the characteristics associated with the static nonlinearity (N in the LN model) and therefore generalizes to any such nonlinearity supporting a sensible discrimination model. In other words, even if one were to allow for different characteristics of the static nonlinearity to be associated with different noise types, the 2D LN model just considered would not be compatible with our results.

The structural failure detailed above can only be addressed by inserting a model component orthogonal to the target signal; because peak and trough amplitudes were comparable (Fig 2H), it would appear that the orthogonal component should be assigned equal gain to the component aligned with target orientation. We must rule out a push-pull model where the template is obtained by simply taking the difference between target and non-target signals (we further consider this model later in the article), because the associated 2D filter estimate should be itself an image of that difference, contrary to the observed PF (Fig 2A and 2E–2F). The next minimal incremental modification of the push-pull model involves squaring the output of the two templates before they are subtracted [51] (Fig 5F). This variant, which no longer belongs to the LN family, generates orientation-tuned filters fully overlapping with those observed experimentally (compare red and black traces in Fig 5D and 5I), however it fails to generate suitable 2D filter descriptors (Fig 5B and 5G). This failure is once again structural: there is complete symmetry between vertical and horizontal orientations at the level of stimuli, task and model, so that all PF estimates must be similarly symmetric; we observed symmetry for orientation-tuned PF’s (Fig 2H) but not for 2D filters (Fig 2F), ruling out the nonlinear push-pull model. To summarize, the push-pull model improves on the LN model in its superior ability to capture orientation-tuned PF’s (blue data points in Fig 5J fall below the unity line at p < 0.0002 by two-tailed paired WSR test for x different than y values), however it is poorer at accounting for 2D/1D PF’s (red/orange data points in Fig 5H fall above the unity line at p < 10^−4^).

An additional canonical operation in the construction of small-scale circuits for neural computing is divisive normalization [19, 52, 53]. We implemented the most basic version of this operation: the linear drive is supported by the target-like matched template, and the gain-control operator pools from only parallel and orthogonal filters (Fig 6A). This minimal version of gain-control is sufficient to account, at least qualitatively, for all empirical PF estimates with no identifiable structural failure (Fig 6). On average, the model accounts for 94% of the variance in the aggregate data across 1D, Θ and SF conditions (variance accounted for in the 2D condition is inevitably low at 0.27 due to the high density of the noise probe, which results in high measurement noise and a sparsely modulated PF; for this condition we rely on the reasonable qualitative match between fitted surfaces indicated by ovals in Fig 6B and 6F). This value is more than satisfactory when one considers that the relevant implementation involves no free parameters and can be deployed without prior estimate of the underlying 2D filter. To summarize, the gain-control model is able to rectify the inadequacy of the LN model in capturing orientation-tuned PF’s (blue data points in Fig 6I fall below the unity line at p < 0.0002), while at the same time retaining its ability to account for 2D/1D PF’s (red/orange data points in Fig 5G scatter around the unity line at p = 0.43) and characteristic features of the SF-tuning data (magenta data in Fig 6I scatter around the unity line at p = 0.9; see also Fig 6E).

Despite its highly nonlinear nature, the gain-control circuit in Fig 6A operates in a manner well approximated by linear templates when projected onto and defined across each of the four dimensions probed by our experiments: Fig 7A plots indices returned by the two LN tests previously applied to the human data (see Fig 3A); there was no detectable sign of departure from template matching for any condition under either test (black/gray histograms centred around 0). This result is not due to lack of resolving power: when applied to the push-pull model (Fig 7B) both tests return positive distributions for the 1D condition, because this model generates mismatched target-present and target-absent estimates (compare red with blue traces within left inset to 1D condition in Fig 7B; see also clear modulations of its second-order kernel, right inset). Neither test detects departures from LN transduction for the 2D condition, however this is not because the PF’s associated with the push-pull model were consistent with template matching, but rather because they were nearly featureless and therefore inconsistent with the human data (2D orange distribution in Fig 7B, reflecting first-order PF sructure, is centred around 0; see also Fig 5B and 5G).

As a final step in cross-checking the applicability of the gain-control model, we computed its trial-by-trial predictive power and confirmed that it falls within the optimal range (inset to Fig 4B): collectively across all estimates, human-model agreement falls significantly above/below the lower/upper bounds of the maximum predictability region (p < 10^−6^/10^−8^) and does not differ from the midpoint between the two bounds (p < 0.42).

### Engagement of multiple elementary operators

The above detailed experiments were specifically designed with the objective of isolating a single elementary operator for cortical processing of visual signals. One stimulus feature associated with this effort involved placing two vertically oriented signal templates above and below the probe region (see Methods and S1 Video). As explained previously, these templates served the purpose of prompting a matching strategy on the part of the human observers [48]; if observers could not implement a pure matching strategy (as supported by our results), the target templates would at least prompt reliance on the read-out mechanism associated with the target signal, whether the horizontally oriented non-target signal was absent (detection) or present (discrimination). In this manner, we hoped to facilitate experimental conditions where observers relied on the same elementary operation across different tasks, so that we could inspect the properties of said elementary operation in the presence of different stimulus conditions. The similarity between PF’s derived from the two tasks (detection versus discrimination) provides compelling evidence that we succeeded in this targeted effort.

In the discrimination task, the optimal strategy for observers is to engage the difference between the elementary operator associated with extracting the vertically oriented target signal and the elementary operator associated with extracting the horizontally oriented non-target signal. However, because we deliberately steered observers towards relying only on the former operator (while ignoring the latter) via placement of the target templates, it is conceivable that the observed departure from the optimal strategy (Fig 2E–2F) may be a direct consequence of our targeted effort to bias their read-out machinery towards the just detailed suboptimal strategy. It may therefore be of interest to characterize the system under conditions in which the combined output of the two elementary operators is facilitated by the additional placement of non-target templates to the sides of the central probe region (Fig 8A, green).

Although both 2D and orientation PF’s associated with this ‘symmetric’ variant of the discrimination task largely resemble those obtained in the absence of the non-target templates (compare Fig 8B and 8C with Fig 2E and 2G), there are important differences. First, the symmetric variant is associated with a measurable presence of the horizontal component. Although this effect is only mildly visible at the level of the aggregate 2D PF (Fig 8B, left), it is more clearly exposed by the non-target match values obtained from individual observer PF’s. More specifically, all 5 observers who had not participated in the original version of the discrimination task (that is in the absence of non-target templates) returned negative match values (all black data points in Fig 8D fall to the left of the vertical dashed line), indicating the presence of the negative non-target image expected under conditions where both vertical and horizontal elementary operators are engaged. This result was not observed for the original variant of the discrimination task (open symbols in Fig 2F). The only observer who did not follow the pattern prompted by the new variant of the discrimination task (grey data point in Fig 8D) was also the only observer from this pool who had participated in the original variant; it is reasonable to interpret this finding as reflecting the possibility that this observer retained the read-out strategy she had previously developed and failed to readjust.

A second important feature that characterizes the symmetric variant of the discrimination task is the appreciable difference between target-present and target-absent PF’s. Qualitative inspection of the target-present PF (Fig 8B, top right) suggests that it contains primarily if not exclusively vertical structure, while the target-absent PF (Fig 8B, bottom right) resembles more closely the difference between vertically-oriented and horizontally-oriented operators. These qualitative impressions are quantitatively confirmed by the lack of significant non-target content within target-present PF’s from individual observers (red data points in Fig 8D scatter around the vertical dashed line) and by the roughly equivalent content of target and non-target match within target-absent PF’s (blue data points in Fig 8D scatter around the diagonal negative unity line). Orientation-tuned PF’s are suggestive of potentially related differences: although these effects appear slight upon qualitative inspection of aggregate PF’s (compare red versus blue traces in Fig 8C), target-absent PF’s from individual observers present less marked peak values (corresponding to the target orientation) and more marked trough values (corresponding to the non-target orientation) than target-present PF’s (blue data points are shifted down and to the left of red data points in Fig 8E).

These differences between target-present and target-absent PF’s are indicative of a departure from the LN model [21, 31–38]. We therefore expect that this departure should be measurable using the first-order/second-order nonlinear tests we have applied to previous data. Indeed, we not only find that taken together the two tests return significantly positive values (at p < 0.002), but also that they strongly correlate (green data points in Fig 8I extend into the upper-right quadrant and display correlated scatter at r = 0.77, p < 0.004). No such effects are visible for the original variant of the discrimination task (black data points in Fig 8I).

We attempt to model these results by combining the modules developed in the previous section. The primary goal of this exercise is to exclude a role for template matching, not to simulate all details of the dataset. For this reason, we do not attempt to capture the observed differences between target-present and target-absent PF’s. The LN model involves subtracting the output of the horizontal template matcher from the output of the vertical template matcher. Because the empirical 2D PF contains more vertical than horizontal structure (Fig 8D), we halved the output of the horizontal template matcher before applying the subtraction in order to improve its ability to simulate the 2D condition (see Methods). For this model, we know that target-present and target-absent PF’s do not differ [21, 31–38] (not shown). The output reduction applied to the horizontal component translates into a reduced trough within the orientation-tuned PF (magenta in Fig 8G). If we do not apply output reduction, the LN model successfully returns trough and peak of equal amplitude for the orientation-tuned PF as observed experimentally, but fails to capture the unbalanced structure of the 2D PF. In other words, the LN model is able to account for either condition (2D versus orientation) in isolation, but not both concomitantly (similar to what we found previously).

The gain-control model fared substantially better. Again, we subtracted a horizontally-tuned gain-control circuit from the vertically-tuned gain-control circuit developed previously (Fig 6A). As with the LN model, the output of the horizontally-tuned circuit was halved before subtraction. This model captures both 2D and orientation-tuned PF’s, in that it produces trough and peak of equal amplitude and overlaps fully with the human data (Fig 8F–8G). Although it is therefore superior to the LN model (Fig 8H), we find that it does not generate appreciably different target-present versus target-absent PF’s (top/bottom right in Fig 8F), failing to account for this specific feature of the human data. It is conceivable that this failure may be ameliorated by more elaborate variants of the gain-control model, however this is not our goal. As we have explained above, due to its mixed nature, the read-out mechanism engaged by observers in the symmetric variant of the discrimination task is not ideal for achieving the goal of excluding/supporting the LN model using a non-parametric approach. Although it is certainly interesting to consider this variant and how it may lead to partially different results, our core conclusions rely on the main detection/discrimination tasks; under those conditions, a single elementary operator can be characterized in isolation.

## Discussion

### The many attractions of template matching

It is uncontroversial that human vision often displays highly nonlinear characteristics [54], yet the linear-nonlinear model retains a paramount role in shaping past and current accounts of this fundamental sensory process. There are at least two reasons for its popularity.

First, the presence of an output static nonlinearity combined with a judicious choice of input space for stimulus projection often allow for effective and compact accounts of apparently more complex phenomena. A fitting example is motion detection, an inherently nonlinear process [55]. In the retina, this phenomenon is typically modelled using Reichardt cross-correlation [56], a nonlinear scheme that combines the output of multiple (at least two) elementary units [57]. Although this model does not conform to the LN scheme with reference to its original structure, it can be recast in the form of a linear oriented spatiotemporal filter followed by a static nonlinearity [58]; indeed, the latter scheme is more commonly adopted to account for cortical and behavioural processes [59, 60].

Second, LN models are often adequate for qualitative thinking and descriptive purposes, particularly when the system is challenged under a limited range of experimental conditions. This approach is exemplified by popular accounts of contrast-based illusions like the Hermann grid phenomenon [61], where the associated phenomenology is referred back to LN models incorporating front-end linear filters with an inhibitory surround [11]. Under some conditions, these accounts can be exploited to some degree of quantitative interpretation, providing for example a rationale for the broad agreement between filter size estimated from the above model of the Hermann grid illusion, and corresponding neuronal receptive field size measured electrophysiologically at ranging eccentricities [62].

Practically speaking, the LN model is attractive thanks to its analytical tractability. Combined with controlled input stimulation, this model makes simple predictions that have been extensively exploited to support characterization of the front-end linear filter [4, 16, 63]. Indeed, transparent interpretability of most measurements presented in this study is largely compromised wherever the LN model becomes inapplicable, although we have demonstrated here and in previous work that adequate tools exist for tackling such situations [10].

It is therefore clear that LN models are both useful and desirable, but it is also clear that they may fail under a range of conditions for quantitative purposes. We currently have no clear indication of when such failures may occur, how widespread they may be, and to what extent they may impact quantitative conclusions regarding human sensory processing. Only a few studies have examined this issue in sufficient detail [26, 36, 37, 44], and none has carried out an extensive characterization that would deliver a comprehensive picture across substantially different aspects of the same stimulus/task. As we discuss below, an integrated approach of the latter kind does not merely represent a quantitative extension of previous efforts, but rather enables a qualitatively different level of dissection of the relevant mechanisms and supports novel conclusions not available to previous studies.

### Why template matchers succeed at one level, but fail at a deeper level

The present study represents an attempt to determine whether there is at least one limited set of identifiable conditions under which human vision engages exclusively LN circuits. It is not intended as an attempt to determine whether the entirety of human visual processing can be reduced to LN transduction: as indicated above, an attempt of this kind would be fool-hearted, because it is inconceivable that the whole of vision would involve no more than template matching. Rather than asking whether all visual operations are template matchers, we ask whether there is at least one visual operation that involves template matching. We reasoned that the strongest and most relevant test of the latter possibility would involve task and stimulus specifications that are not only representative of core interests in vision science [43], but also probe elementary operations supported by visual cortex [64] using experimental protocols specifically designed to facilitate LN transduction (see below for further discussion of these points).

There is a sense, of considerable practical significance, in which our results provide encouraging evidence to support the applicability of LN models for understanding and characterizing human pattern vision: under all the experimental conditions we tested, the human process could be adequately described in the form of a simple LN model applied to the dimension probed by the perturbation associated with a given noise mask. This result enables a wide range of tools that have been tailored to the LN family [4, 16, 65]. It must be emphasized that the conditions of the detection/discrimination experiments presented here were carefully adjusted to prompt a template-matching strategy on the part of human participants. As we have demonstrated with the symmetric variant of the discrimination task (Fig 8), apparently irrelevant methodological details (e.g. inclusion of target/non-target replicas adjacent to the probe) may impact the extent to which these experiments are representative of a wider range of specifications: it goes without saying that, as more elementary operators and processing layers are loaded onto the read-out stage, the collective process (which in the limit will encompass the whole of vision) will inevitably manifest departures from LN transduction.

There is however a different sense, arguably of greater theoretical significance, in which our results are not equally supportive of LN modelling schemes: it is the sense of understanding the deeper structure of the system beyond its superficial compliance with LN transduction in the manner discussed above (see [63] for related pursuits in neuronal modelling). In this sense, we were unable to identify a single implementation of a specific LN model that would capture all aspects of our complex dataset (Fig 5; see related results from electrophysiological recordings of simple cells [24, 53]). This failure was structural in that it involved qualitative departures from the human data that could not be ameliorated via further exploration of parameter space. Outside the LN family, we successfully identified a viable candidate by incorporating a canonical computation for cortical circuits: gain control via divisive normalization [19]. The applicability of this operation to neural processes is extensively documented [52, 66]. The success of this model in capturing our own data therefore conforms to current trends in the computational literature. The non-parametric logical/analytical process by which we mustered support fo the gain-control model differs in its outlook from previous attempts based on fitting multiparametric models [67–70]; nevertheless, it is notable that gain control circuits feature prominently in those studies too.

Is it conceivable that a comparable set of experiments might have been identified that did not require any modelling tools outside the LN family? This seems unlikely. As mentioned earlier, extensive piloting went into ensuring that template matching would be encouraged as exhaustively as feasible on the part of human participants, leaving little room for further tailoring of stimulus specifications to favour LN strategies. Furthermore, the function probed by our protocol is elementary: Gabor wavelets represent some of the most efficiently detectable visual patterns [64], consistent with the notion that their structure resembles neuronal preference in cortex [71]. Any detection/discrimination task relevant to human visual function would presumably involve combinations of analogous operations [72, 73], leading to the expectation that it would display at least an equivalent, if not more pronounced, degree of departure from the LN model. This is indeed the result we observed when we altered the discrimination protocol to prompt a strategy whereby observers would combine two elementary operations (Fig 8). Based on the above considerations, we believe that our experiments are ideally positioned to draw conclusions about the applicability of LN models to human vision: if there exist any conditions, no matter how limited, under which such models are applicable, those conditions would include the specifications probed by our protocols.

The outcome of our experiments enables one characterization for the underlying mechanism while excluding a number of alternative scenarios, all nonetheless viable and plausible. In other words, it would have been entirely reasonable to expect and observe a substantially different outcome. Under one scenario, the orientation and SF tuning characteristics returned by our PF measurements may have conformed to those predicted by the specified 2D version of the LN model (blue traces in Fig 5D and 5E), leaving open the possibility that the system was exclusively engaging LN circuits across the board. Under a different scenario, the system may have operated in the manner of the push-pull model outlined in Fig 5F (see [51] for a concrete example of the applicability of this model to data from a discrimination task closely related to the one used here): lack of compliance with LN transduction would have then become apparent from applying the linearity tests to one condition alone (1D, see Fig 7B), yielding the conclusion (opposite to the one immediately above) that the system was engaging more elaborate circuitry than LN components, and furthermore that the design of such circuitry did not support LN behaviour even when restricted to individual probes (as we observed for the symmetric variant of the discrimination task, see Fig 8D–8E and 8I). Both our findings, i.e. that system structure does not conform to LN circuitry and yet complies with LN transduction under varying conditions, are therefore independent contributions that place important constraints on the range of plausible scenarios potentially associated with the visual processes examined here. It is relevant in this respect that our ability to dissect the underlying mechanisms with adequate discriminatory power specifically relied on evaluating different features of our combined dataset: had we considered each condition in isolation (e.g. only the 2D results or only the orientation-tuning results), it would have been impossible to constrain our conclusions to the extent that was enabled by the integrated analysis presented here.

### A nonlinear wolf in linear sheep’s clothing

**Fig 9:**
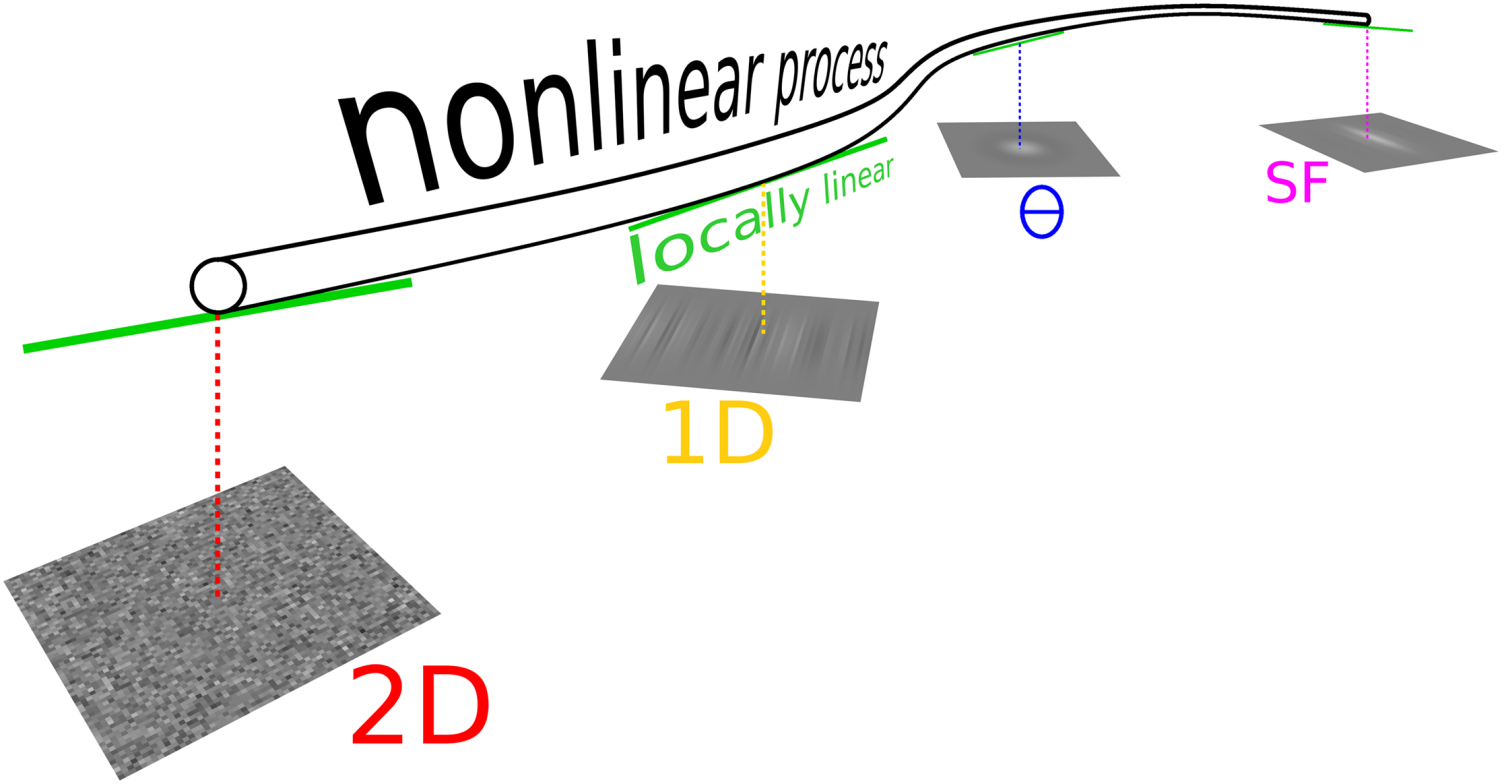
The sense in which vision is linear, and the sense in which it is not. Our results can be intuitively summarized with relation to the simple notion of piecewise linear approximation to a nonlinear function, although this parallel should not be taken literally: the sensory process is far more complex than can be represented here using a single trajectory. It is nevertheless useful to imagine this process as the pictured 3D pipe-like structure, spanning all 4 different spaces within which we defined and perturbed the visual stimuli. When the process is probed within each individual stimulus space, its characteristics can be adequately approximated by a linear model. A comprehensive account of its collective properties spanning all 4 spaces, however, cannot be achieved using a linear approximator: the system may appear linear every time it is probed from a different direction, yet its underlying structure remains highly nonlinear.

A potentially productive way of summarizing our results may be obtained via reference to the simple concept of locally linear approximation for nonlinear functions. As illustrated by the cartoon in Fig 9, we can think of the visual process as a manifold spanning a space that encompasses all possible dimensions across which the stimulus may be usefully represented (clearly such high dimensional spaces cannot be adequately represented in a 2D cartoon, so Fig 9 is only intended as an intuitive tool and not an accurate description of the process). When projected onto a specific subspace (e.g. orientation or spatial frequency), and when inspected with respect to that restricted subspace, the behaviour exhibited by the process may be satisfactorily approximated by the LN framework, even though this framework may not be adequate to describe the process as a whole, i.e. with respect to its collective characteristics across multiple projections. Our results indicate that the operations of human vision, no matter how elementary and limited in their immediate scope (e.g. detection of a Gabor wavelet), cannot be reduced to a straight pipe through Fig 9: they retain an irreducible level of nonlinear structure possibly reflecting the minimal functional characteristics implemented by cortical circuits [5, 19, 52, 53].

Fig 9 may misleadingly suggest that the introduction of different noise masks in our study is equivalent to the expansion of stimulus range afforded by previous investigations that manipulated e.g. stimulus uncertainty [20, 21, 37] and/or pedestal contrast [67, 74] (these two factors being potentially intertwined [20, 74–76]). From the perspective of those and related studies, it may seem trivial that linearity breaks down as stimulus range is expanded. There is however a critical difference with respect to our approach, in that most previous studies manipulated the range/specification spanned by the signal to be detected, thereby potentially prompting the system to engage different modules/regimes to perform the assigned task [36]. The target signal to be detected in our experiments was fixed and uniquely specified, regardless of the noise perturbation that was added to it. Under these conditions, observers were prompted to engage a single elementary perceptual operator. Furthermore, the effects of expanding stimulus range have often been modelled via LN cascades with a sigmoidal static nonlinearity and/or one that changes exponent [13, 74, 77], but which nevertheless perform LN transduction; our results exclude LN models regardless of the specific characteristics associated with the static nonlinearity, and regardless of whether those characteristics may differ for different noise types. Finally, the notion that linearity should break down as stimulus range is expanded implicitly relies on the assumption that linearity does apply in the first place within a restricted input range; this assumption has never been adequately checked, at least not to the extent afforded by the experiments/analyses presented here. With these caveats in mind, Fig 9 is best interpreted *not* as the trajectory of a perceptual system that traverses different stimulus regimes and potentially modifies its characteristics along the way, but rather as the intrinsic structure of an elementary process operating within a minimally defined input range. The combined application of different noise probes delivers a multifaceted view of this process that is not afforded by each individual probe in isolation.

It may seem counterintuitive that an inherently nonlinear architecture would be in place for it to behave linearly with respect to substantially different visual dimensions, such as 1D space or orientation as probed by our stimuli. If linear transduction is the goal, why not implement it using the template matcher in Fig 5A? If conversely nonlinearity is the goal, why build a nonlinear system that retains such degree of linear transduction as we measured here, and not adopt the push-pull circuit in Fig 5F regardless of its highly nonlinear transduction properties (Fig 7B)? Our results suggest that, at least under the conditions of our experiments, the system strives to achieve linear encoding: it seems otherwise difficult to explain why we observed such extensive compliance with template matching for processing 4 different noise probes across 2 separate tasks, when prior studies have exposed clear deviations under more limited conditions [4, 21, 34, 36–38].

Although elementary template matchers like the mechanism in Fig 5A support linear transduction, they may not be adequate for the purpose of versatile stimulus encoding in ways that are both linear and useful; in this context, utility may involve the necessity to represent orientation in a balanced push-pull fashion as we observed in our experiments, a goal that cannot be achieved by the circuit in Fig 5A (see blue traces in Fig 5D and 5I). An alternative strategy, supported by our findings, involves assembling small nonlinear circuits that support effective stimulus encoding (e.g. push-pull orientation selectivity as in Fig 2C and 2G and sharp bandpass SF tuning as in Fig 2D) while at the same time retaining linearity across a wide range of tasks and stimulus perturbations [24, 52]. Divisive normalization has proven an effective tool for efficient transduction while maintaining gain within near-linear regimes [19]; Fig 9 elaborates on this property to encompass the collective space of extended stimulus projection for multi-feature encoding (with the caveats outlined above). Although this interpretation is highly speculative, its proposed mode of operation is known to underlie other aspects of sensory processing [66, 78], in particular retinal encoding of ON/OFF signals: in the retina, linearity is a luxury that comes at the cost of carefully assembled nonlinear subunits [79]. Our results suggest that similar principles may apply to some cortical computations [80].

## Supporting Information

S1 Video Examples of stimuli used in the experiments, looping through 2D, 1D, orientation and SF noise probes in this order from trial to trial (each trial consists of two intervals). (SWF)

S1 Text Baseline index of trial-by-trial coupling between input perturbation and behavioural response. (TEX)
